# Author Correction: Behavioural individuality in clonal fish arises despite near-identical rearing conditions

**DOI:** 10.1038/s41467-026-72301-2

**Published:** 2026-05-05

**Authors:** David Bierbach, Kate L. Laskowski, Max Wolf

**Affiliations:** Department of Biology and Ecology of Fishes, Leibniz Institute of Freshwater Ecology and Inland Fisheries, Müggelseedamm 310, 12587 Berlin, Germany

Correction to: *Nature Communications* 10.1038/ncomms15361, published online 17 May 2017

After being alerted by commenters on PubPeer, we identified an error in the body size data we initially used for our analyses: body size values were inadvertently assigned to the wrong individuals. From our documentation, we retained the original body size data and re-ran our analysis with the corrected data. While there were no changes in our main findings or conclusions drawn, we here report the corrected analyses.

As in the original manuscript, there was no evidence for differences in body size among treatments (0-day treatment size estimate: 23.61 mm with 95% CI [22.58, 24.64]; 7-day treatment: 23.18 mm [22.14, 24.20]; 28-day treatment: 22.67 mm [21.65, 23.77]), or interactions between treatment and body size (inclusion of the interaction did not improve model fit ΔDIC = +1.29; where smaller DIC values are considered better fit) or treatment and observation (inclusion of the interaction did not improve model fit ΔDIC = +2.11). The corrected analysis reveals a slight effect of body size on behaviour smaller fish are more active than larger fish (see updated Table 1 in Supplementary Information).

Importantly, however, inclusion of the corrected body sizes does not alter our main findings and conclusions drawn: (i) substantial individual variation in behaviour emerges among genetically identical individuals isolated directly after birth into highly standardized environments and (ii) increasing levels of social experience during ontogeny do not affect levels of individual behavioural variation. The corrected data along with our analysis code have been uploaded to Dryad (see 10.5061/dryad.td3sj or ref. 58 in the original article) and can be used to re-run our analysis.

Due to its age, the article cannot be updated directly. A list of corrections and a revision to the original Supplementary Table 1 are available in the Supplementary Information accompanying this amendment.

## Supplementary information

List of edits to original article; revised Supplementary Table 1

