# Supplementary material for: Behavioural individuality in clonal fish arises despite near-identical rearing conditions
Source: Nat Commun. 2017 May 17;8:15361. doi: 10.1038/ncomms15361 (PMC5442312; doi:10.1038/ncomms15361)
Supplement: Supplementary Information — Supplementary Table [file ncomms15361-s1.pdf]

Supplementary Table 1. Comparison of models with different random (co)variance structures. Within each treatment, we compared the fit of models with no random effects, random intercepts, and random intercepts and slopes across both individuals and mothers; we consider a change in DIC score of greater than 3 to indicate a significant difference in model fit. In all treatments, the models with only random intercepts at the Individual were best supported as the most parsimonious model with the lowest DIC score; however because of the hierarchical nature of the experimental design (Individuals nested within Mother) we decided to retain terms for intercepts at both the Individual and Mother level (bolded scores). The random structure is written in the syntax of MCMCglmm R package.

Random structure	0-day	7-day	28-day
no random effects	856.36	819.88	824.05
~ ID	831.28	786.48	806.65
~ ID + Mother	**831.69**	**784.91**	**806.59**
~ idh(1+obs):ID + Mother	830.15	785.78	806.96
~ idh(1+obs):ID + idh(1+obs):Mother	830.35	786.79	808.24
