# Supplementary material for: Author Correction: Behavioural individuality in clonal fish arises despite near-identical rearing conditions
Source: Nat Commun. 2026 May 5;17:4065. doi: 10.1038/s41467-026-72301-2 (PMC13144405; doi:10.1038/s41467-026-72301-2)
Supplement: Supplementary file 1 — List of edits to original article; revised Supplementary Table 1 [file 41467_2026_72301_MOESM1_ESM.pdf]

Supplementary Information to Author Correction: Behavioural individuality in clonal fish arises despite near-identical rearing conditions

Nature Communications https://doi.org/10.1038/ncomms15361, published online 17 May 2017.

Corrections made to original article

Changes are highlighted in yellow and continuously numbered, showing first the original section of text, followed by the corrected one.

1. Page 3, Table 1:

ORIGINAL: Table 1:

**Fixed effects**	**Estimate (95%CIs)**		
Standard length	0.12 (-0.37, 0.61)		
Observation	2.63 (2.11, 3.13)		
Treatment			
0-day	7.79 (-4.00, 18.72)		
7-day	7.41 (-4.58, 17.73)		
28-day	9.08 (-2.17, 20.60)		
**Variance estimates**			
Among-mother	3.89		
	**0-day**	**7-day**	**28-day**
Among-individual variance	19.36 (5.28, 36.28)	17.99 (3.34, 34.83)	11.35 (1.59, 22.61)
Within-individual variance	39.35 (28.37, 51.39)	30.88 (22.23, 39.98)	26.21 (19.11, 34.36)
Repeatability	0.35 (0.13, 0.52)	0.35 (0.13, 0.57)	0.29 (0.09, 0.51)

CORRECTED: Table 1

**Fixed effects**	**Estimate (95%CIs)**		
Standard length	-0.83 (-1.42, -0.22)		
Observation	2.65 (2.14, 3.18)		
Treatment			
0-day	30.05 (15.02, 43.65)		
7-day	29.38 (15.30, 43.45)		
28-day	29.86 (16.35, 43.80)		
**Variance estimates**			
Among-mother	2.77 (0, 8.83)		
	**0-day**	**7-day**	**28-day**
Among-individual variance	17.02 (3.48, 32.33)	13.55 (1.86, 26.68)	10.76 (2.40, 20.88)
Within-individual variance	39.44 (28.05, 51.10)	31.11 (22.56, 40.74)	26.14 (19.23, 34.06)
Repeatability	0.31 (0.11, 0.50)	0.29 (0.09, 0.51)	0.29 (0.09, 0.47)

2. Page 3, left column:

ORIGINAL: “Indeed, the model containing treatment-specific variance estimates was not better supported than a model where individual variance was constrained to be the same across all three treatments (ΔDIC= +0.667),…”

CORRECTED: “Indeed, the model containing treatment-specific variance estimates was not better supported than a model where individual variance was constrained to be the same across all three treatments (ΔDIC= +1.07),…”

3. Page 3, left column:

ORIGINAL: ”Additionally, the inclusion of mother identity as a random effect was not well supported (ΔDIC= -0.445) and…”

CORRECTED: ”Additionally, the inclusion of mother identity as a random effect was not well supported (ΔDIC= -0.57) and…”

4. Page 3, left column:

ORIGINAL: **“**Furthermore, there were no differences in overall activity levels (treatment estimates in Table 1) or in average body size (0-day: 22.89 mm, 95% confidence interval: (21.75, 24.12); 7-day: 22.34 mm (21.11, 23.51); 28-day: 22.70 mm (21.52, 23.90)). We note that…”

CORRECTED: **“**Furthermore, there were no differences in overall activity levels (treatment estimates in Table 1) or in average body size (0-day: 23.61 mm, 95% confidence interval: (22.58, 24.64); 7-day: 23.18 mm (22.14, 24.20); 28-day: 22.67 mm (21.65, 23.77)). However, there was a small but significant effect of body size on behavior - smaller fish were slightly more active than larger fish. Our analysis did not show any significant interactions between treatment and body size (ΔDIC = +1.29) or treatment and observation (ΔDIC = +2.11). We note that…”

5. Page 4, figure 2:

ORIGINAL:

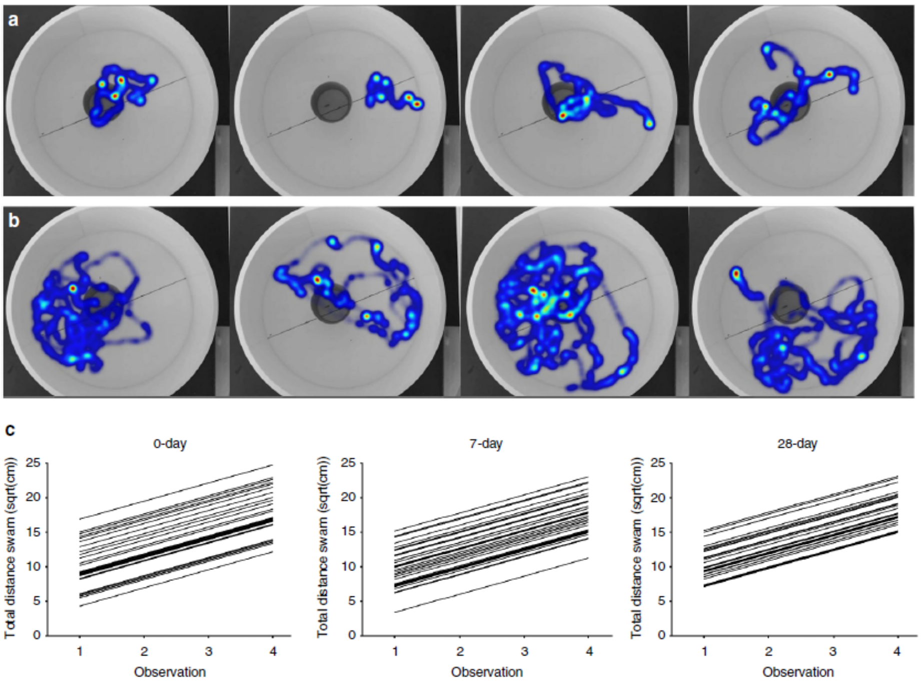

CORRECTED:

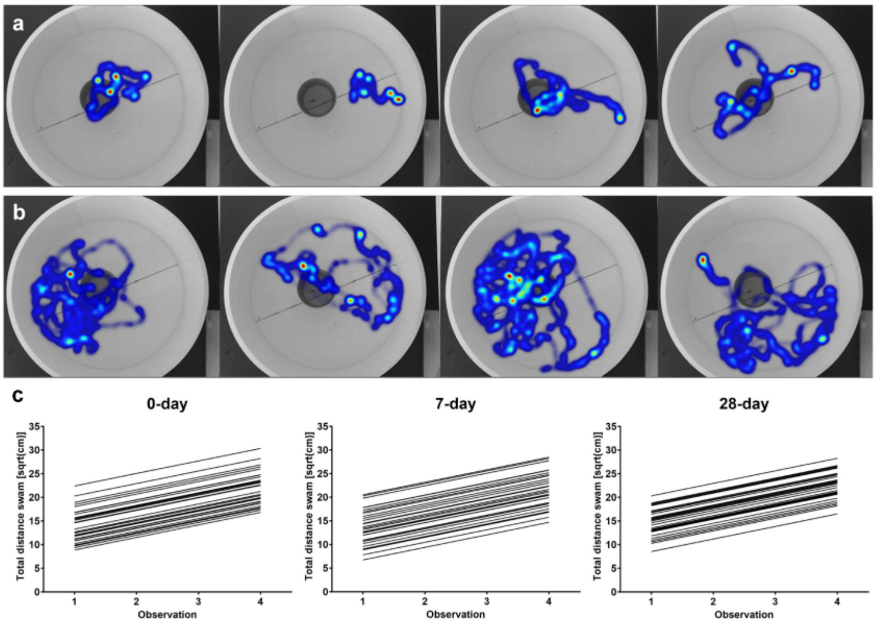

6. Page 5, right column, last sentence of first paragraph from "Statistical analysis.”**:**

ORIGINAL: “Additionally, there was no evidence for an interaction between treatment and body size, or treatment and observation (data not shown).“

CORRECTED: “Additionally, there was no evidence for an interaction between treatment and body size, or treatment and observation (see Results section).”

7. Page 5, right column, data availability statement:

ORIGINAL: Data availability. All data are accessible via dryad respository[58].

CORRECTED: Data availability. All data and R-code are accessible via dryad respository[58]

8. Supplementary Table 1 has been corrected and the original and corrected Supplementary Table 1 are deposited along with the manuscript.

9. Dryad: The following files are now available at Dryad at https://doi.org/10.5061/dryad.td3sj

1. Clonal_molly_behav_indv_statistical_code_SL_CORRECTED.Rmd - actual code that can reproduce our result ()
2. Clonal-molly-behav-indv-statistical-code_SL-CORRECTED.pdf - pdf with our code and all the results
3. Clonal_molly_behavioral_individuality_for_deposit_SL_corrected.csv - the CORRECTED data file (including both the correct and wrong SL data)

Supplementary Table 1

Comparison of models with different random (co)variance structures. Within each treatment, we compared the fit of models with no random effects, random intercepts, and random intercepts and slopes across both individuals and mothers; we consider a change in DIC score of greater than 3 to indicate a significant difference in model fit. In all treatments, the models with only random intercepts at the Individual were best supported as the most parsimonious model with the lowest DIC score; however because of the hierarchical natural of the experimental design (individuals nested within Mothers) we decided to retain terms for intercepts at both the Individual and Mother level (bolded scores). The random structure is written in the syntax of the MCMCglmm R package.

Random structure	0-day	7-day	28-day
No random effects	852.35	809.72	824.16
~ ID	829.58	783.57	803.43
~ ID + Mother	**830.04**	**783.47**	**803.85**
~ idh(1+obs):ID + Mother	829.03	784.63	804.85
~ idh(1+obs):ID + idh(1+obs):Mother	829.34	785.47	806.02
